# Beyond the Rotisserie: Dispelling the Myths of Intravesical Therapy and Pioneering a Precision Future for Bladder Cancer

**DOI:** 10.5152/tud.2026.25112

**Published:** 2026-05-15

**Authors:** Mohammad Z. Khan, Mohamed A. Atta, Aly Kotb, Asmaa Ismail, Ahmed Kotb

**Affiliations:** 1 Department of Surgery, California University of Science and Medicine, Colton, USA; 2 Department of Urology, Alexandria University, Alexandria, Egypt; 3 Department of Surgery, Grande Prairie Regional Hospital, Alberta University, Grande Prairie, Canada

**Keywords:** Bacillus Calmette-Guérin vaccine, intravesical administration, patient positioning, precision medicine, urinary bladder neoplasms, urinary catheterization

## Abstract

In the treatment of intermediate and high-risk non–muscle-invasive bladder cancer (NMIBC), intravesical therapy with Bacillus Calmette–Guérin or chemotherapy remains the gold standard. However, traditional methods involving patient repositioning and catheter retention lack robust evidence. A focused narrative review of international guidelines (European Association of Urology, American Urological Association, Canadian Urological Association) and PubMed-indexed literature was conducted to address the rationale, efficacy, and pharmacokinetics of intravesical delivery, with particular attention to patient rotation and catheter management. Available evidence does not demonstrate a clinically meaningful benefit of routine post-instillation patient rotation for intravesical therapy. Major international guidelines do not recommend routine patient rotation during intravesical dwell time. Dwell times beyond 1 hour show diminishing incremental benefit, and prolonged catheter retention may limit uniform urothelial contact. Modeling studies suggest increased volume may enhance mucosal penetration but must be balanced with patient tolerance. Contemporary practices increasingly support immediate catheter removal following instillation. Emerging data suggest that tumor grade and biological features may complement traditional stage-based risk stratification. It was concluded that routine patient repositioning during intravesical treatment dwell time is not supported by evidence; no scheduled turns are required, thereby improving comfort and adherence. Immediate catheter removal after instillation may optimize urothelial exposure and improve patient tolerability, while potentially reducing catheter-associated complications. Clamped Foleys should be reserved for patients unable to voluntarily retain fluid and should be removed at the earliest opportunity. Advances in tumor grading, molecular characterization, and cautiously applied artificial intelligence tools may further refine risk stratification and personalize intravesical therapy in the future.

**Main Points**

- Routine patient repositioning (“rotisserie” protocols) during intravesical therapy is not supported by clinical evidence and is not recommended by major international guidelines.
- A dwell time of approximately 1 hour provides effective urothelial exposure, with diminishing benefit beyond this duration and no demonstrated need for enforced patient rotation.
- Immediate catheter removal after intravesical instillation may improve patient comfort, reduce catheter-associated complications, and does not compromise therapeutic efficacy.
- Clamped Foley catheters should be reserved for select patients unable to voluntarily retain intravesical agents and removed as early as feasible.
- Advances in tumor grading, molecular profiling, and emerging analytic tools may complement traditional stage-based risk stratification and support a more personalized future for intravesical therapy.

## Introduction

Non–muscle-invasive bladder cancer (NMIBC) accounts for approximately 70% of newly diagnosed bladder tumors and is characterized by substantial rates of recurrence (30%-50%) and progression (10%-20%) following transurethral resection of bladder tumor (TURBT) alone.^1^ Intravesical therapy—direct instillation of anticancer agents into the bladder lumen—has therefore become a cornerstone of NMIBC management, improving oncologic control while preserving bladder function and patient quality of life. Since Morales et al first introduced intravesical Bacillus Calmette–Guérin (BCG) in 1976, multiple randomized trials and meta-analyses have demonstrated its superiority over TURBT alone in reducing both tumor recurrence and progression. A landmark European Organization for Research and Treatment of Cancer meta-analysis reported a 27% reduction in invasive recurrence with maintenance BCG compared with TURBT alone (9.8% vs. 13.8%),^2^ while other large studies have documented up to a 40% reduction in 5-year recurrence rates with maintenance therapy.^3^

Intravesical chemotherapeutic agents—including mitomycin-C (40 mg in 40-60 mL), gemcitabine (2000 mg in 100 mL), and valrubicin—serve as primary or salvage options for patients who are intolerant of or refractory to BCG, achieving recurrence reductions of approximately 20%-25% with acceptable toxicity profiles.^4^ Despite the widespread adoption of these agents, considerable variability persists in intravesical administration techniques, including catheter management, instillation volume, dwell time, and post-instillation patient positioning.

This focused narrative review outlines contemporary intravesical instillation practices, including catheter selection, instillation volume, dwell-time management, and biohazard precautions. The historical rationale for systematic patient repositioning—the so-called “rotisserie” protocol—originally intended to promote uniform drug distribution across the bladder mucosa, is examined. Current recommendations from major professional guidelines are synthesized, and the available clinical evidence is critically appraised, including the only randomized trial directly comparing rotation vs. no rotation. Catheter-management strategies and their implications for drug distribution, patient tolerance, and procedural practicality are further evaluated. Finally, streamlined, evidence-informed recommendations are proposed to optimize intravesical therapy in NMIBC while balancing therapeutic efficacy with patient comfort and compliance.

## Methods

This article was designed as a focused narrative review examining the evidence base underlying intravesical therapy techniques for NMIBC, with particular emphasis on patient repositioning, catheter management, instillation volume, and dwell time. The intent was not to perform a systematic review, but rather to critically evaluate the available clinical, guideline-based, and mechanistic evidence informing commonly employed procedural practices.

A structured literature search was conducted to identify clinical studies, guideline documents, and relevant physiological or modeling investigations addressing intravesical delivery techniques. Electronic searches were performed primarily using PubMed/MEDLINE, supplemented by targeted review of major urological society guidelines, including those from the European Association of Urology (EAU), American Urological Association (AUA), Society of Urologic Oncology (SUO), and Canadian Urological Association (CUA). Search terms included combinations of “non–muscle-invasive bladder cancer,” “intravesical therapy,” “BCG,” “mitomycin C,” “gemcitabine,” “patient rotation,” “catheter dwell,” “instillation technique,” “dwell time,” and “drug distribution.” Reference lists of key articles were manually reviewed to identify additional relevant publications.

The literature search focused on English-language publications from 1976 onward, corresponding to the introduction of intravesical BCG therapy into clinical practice. Priority was given to randomized controlled trials, comparative observational studies, guideline statements, and authoritative narrative reviews addressing technical aspects of intravesical delivery. Given the limited number of randomized studies specifically evaluating procedural variables such as patient rotation and catheter management, expert consensus statements, physiologic rationale, and computational or pharmacokinetic modeling studies were included where higher-level clinical evidence was unavailable.

Studies were selected for inclusion based on relevance to intravesical delivery technique rather than oncologic efficacy alone. No formal meta-analysis was performed. Instead, this review aimed to synthesize the available evidence, distinguish data-supported practices from tradition-based techniques, and clarify which aspects of intravesical administration lack empirical validation.

### Epidemiology and Clinical Importance

Non–muscle-invasive bladder cancer comprises approximately 70% of newly diagnosed bladder tumors and represents a substantial proportion of the global bladder cancer burden, with an estimated 550 000 new cases diagnosed worldwide each year.^5^ Despite initial management with TURBT, NMIBC is characterized by high rates of disease recurrence, ranging from 40% to 80% within 5 years, and a clinically meaningful risk of progression, with up to 20% of high-risk cases advancing to muscle-invasive or metastatic disease in the absence of effective adjuvant therapy.^2^^,^^6^

The chronic and relapsing nature of NMIBC imposes a significant economic burden on health systems. In Canada alone, approximately 12 ,500 new cases were diagnosed in 2021, with an estimated per-case direct treatment cost of CAD 658 055 (2011 dollars), translating to over CAD 8 billion in costs for newly diagnosed cases, exclusive of long-term surveillance and recurrence-related care.^7^ Similar cost trajectories have been reported across other healthcare systems, reflecting the need for repeated interventions, lifelong cystoscopic surveillance, and ongoing intravesical treatments.

Beyond economic considerations, NMIBC exerts a sustained negative impact on patient quality of life. Patients frequently report anxiety, discomfort, and treatment-related burden associated with repeated cystoscopies and intravesical instillations, particularly when procedures are perceived as prolonged or physically uncomfortable.^7^ These patient-reported concerns underscore the importance of optimizing not only oncologic outcomes but also procedural tolerability and workflow efficiency.

Within this context, intravesical therapy remains a cornerstone of NMIBC management, and its effectiveness depends not only on agent selection and scheduling but also on the technical aspects of drug delivery. Optimizing instillation technique, while avoiding unnecessary or unsupported procedural practices, represents an opportunity to improve patient experience, enhance adherence, and streamline care without compromising oncologic control. This review therefore focuses on evaluating the evidence underlying commonly employed intravesical delivery practices, particularly those that persist despite limited empirical validation.

### Standard Intravesical Instillation Protocol

Contemporary guidelines from the EAU, AUA, and CUA describe a standardized approach to intravesical instillation, encompassing pre-procedure bladder preparation, instillation of the therapeutic agent, catheter management, dwell time, and post-instillation safety precautions.^8^ Patients are typically instructed to restrict oral fluids for 4-6 hours prior to treatment to minimize residual urine volume, followed by aseptic placement of a lubricated 12-14 Fr catheter and complete bladder drainage.^8^ Therapeutic agents are then instilled slowly over 1-2 minutes to reduce detrusor irritation, most commonly using 120 mg of BCG in 50 mL saline or 20-40 mg of mitomycin-C in 40-60 mL saline.^9^

Instillation volume has been explored primarily through physiologic and modeling studies rather than randomized clinical trials. Computational fluid-dynamic analyses suggest that increasing instilled volume may modestly enhance transmural drug penetration; however, patient tolerability declines substantially at volumes exceeding 75 mL, with minimal incremental benefit observed beyond this threshold.^10^ Accordingly, volumes in the range of 40-60 mL are generally favored in clinical practice, balancing mucosal exposure with patient comfort.

Catheter management following instillation represents another procedural variable with implications for both drug distribution and tolerability. Immediate catheter removal after instillation is widely practiced and supported by expert consensus, as it avoids focal mucosal compression, minimizes catheter-associated microtrauma, and may reduce irritative symptoms and infectious risk. In select patients unable to voluntarily retain intravesical fluid—such as those with severe incontinence or cognitive impairment—a Foley catheter may be temporarily clamped using minimal balloon inflation (typically 2-3 mL). Even in these circumstances, early catheter removal remains preferable once retention is assured.^11^

Dwell time is commonly maintained for 60-120 minutes, depending on agent type and patient tolerance. Historical protocols frequently incorporated structured patient repositioning during this interval, with scheduled 15-minute rotations through supine, prone, and lateral positions intended to enhance intravesical drug distribution.^12^ However, the bladder’s tendency to collapse around low instilled volumes, combined with incidental patient movement during routine activity, appears sufficient to achieve uniform urothelial contact. As such, many contemporary practices permit patients to sit, stand, ambulate, or recline comfortably during the dwell period without enforcing formal rotation.

Following completion of the dwell time, patients are instructed to void—preferably while seated—to minimize splashing. Standard biohazard precautions include instillation of household bleach into the toilet (typically a 1:10 dilution) with a contact time of 15-20 minutes, as well as hand and genital hygiene after voiding for up to 24 hours to reduce exposure risk to caregivers and household contacts.^7^

**Figure 1.**
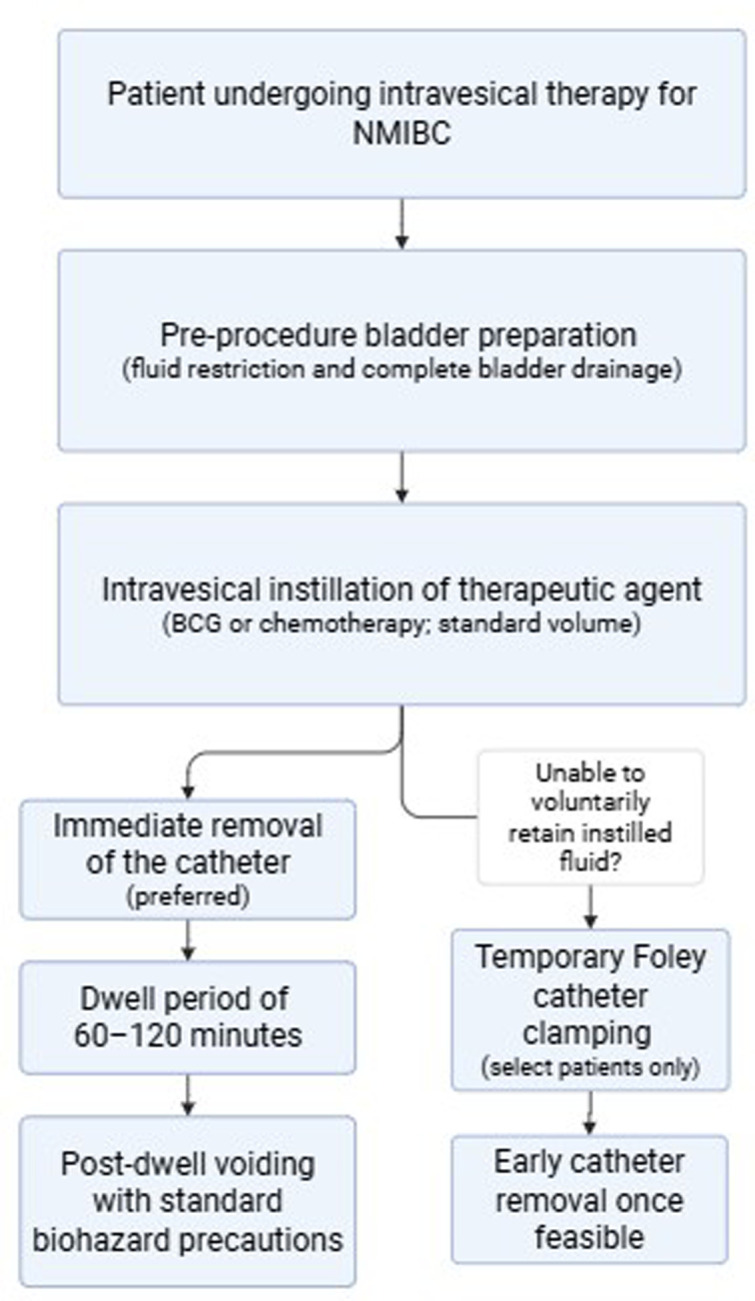
Evidence-aligned intravesical instillation workflow for non–muscle-invasive bladder cancer. Standardized intravesical therapy protocol emphasizing pre-procedure bladder preparation, administration using standard volumes, preference for immediate catheter removal, and a dwell time of 60-120 minutes. Temporary Foley catheter clamping is reserved for select patients unable to retain instilled fluid, with early removal once feasible. Routine patient repositioning during dwell time is not required.

A schematic summary of an evidence-informed intravesical instillation workflow is shown in Figure 1.

### Historical Background: Evolution of Rotation Protocols

The practice of systematic patient rotation during intravesical therapy originated from early concerns regarding uneven drug distribution within the bladder. In their seminal 1976 report describing intravesical BCG, Morales et al^13^ hypothesized that gravity-dependent pooling and intravesical air bubbles could create areas of reduced mucosal exposure, particularly at the bladder dome. To mitigate this theoretical risk, early protocols introduced scheduled patient repositioning, commonly termed the “rotisserie” protocol, whereby patients were instructed to rotate through supine, prone, left lateral, and right lateral positions at 15-minute intervals during the initial dwell period.

Over time, this practice became widely adopted and institutionalized, appearing in nursing manuals, patient instruction sheets, and pharmaceutical package inserts. Notably, the TICE BCG package insert explicitly recommends repositioning patients from side to side and between supine and prone positions to promote uniform mucosal contact. In contrast, the BCG Medac insert advises patients to remain mobile without specifying formal rotation, illustrating early and persistent inconsistencies in manufacturer guidance. Despite its widespread uptake, the rotisserie protocol was incorporated based on physiological reasoning and convention rather than direct comparative clinical evidence.

### Early Expert Critique or Rotation

By the early 2010s, influential clinicians and nursing leaders began questioning the necessity of systematic patient rotation during intravesical therapy. In a 2013 commentary, Shah and Kamat proposed that a collapsed bladder containing approximately 50 mL of instilled solution allows adequate passive fluid–mucosa contact through physiologic wall apposition, thereby rendering formal 15-minute turning protocols unnecessary in routine practice.^12^ They further argued that rotation may only correct a technical error—namely, intravesical air entrapment—and that careful instillation technique, including expulsion of air from the syringe and catheter, largely obviates this concern. Although these expert perspectives challenged long-standing practice, they were based on mechanistic reasoning rather than comparative clinical outcomes, and formal guideline committees appropriately deferred definitive recommendations pending empirical validation.

### Professional Guidelines on Rotation and Instillation

Major urological societies uniformly recognize intravesical therapy as a cornerstone of NMIBC; however, none mandate patient repositioning during dwell time, despite providing detailed technical guidance on dosing, scheduling, and safety considerations.

The EAU NMIBC guidelines (2022-2023) outline recommendations regarding BCG induction and maintenance regimens, instillation duration, and drug concentration, but do not address patient positioning during intravesical dwell time, implicitly reflecting the absence of evidence supporting rotation.^8^ This position is made explicit in the European Association of Urology Nurses (EAUN) guidelines, which conclude that there is no evidence to support repositioning patients to enhance intravesical drug distribution and instead endorse patient-controlled ambulation during dwell periods. The EAUN document also highlights inconsistencies among manufacturer instructions, citing differences between the TICE BCG and BCG Medac package inserts.^8^

Similarly, the AUA NMIBC guideline (2020) omits any recommendation regarding patient rotation, focusing instead on risk stratification, selection of intravesical agents, dosing schedules, and procedural safety.^11^ The AUA educational materials and clinical practice patterns suggest that routine position changes are not enforced in contemporary outpatient settings, reflecting a consensus that such maneuvers lack supporting data.

The CUA guideline likewise provides comprehensive recommendations for intravesical therapy, including induction and maintenance BCG and chemotherapeutic alternatives for BCG-intolerant patients, yet remains silent on post-instillation positioning.^9^ No formal rotational protocols are endorsed within the guideline or its associated publications.

Major international guidelines emphasize evidence-supported technical factors—such as complete bladder drainage to prevent dilution, appropriate instillation volumes, and minimum dwell times for chemotherapeutic efficacy—while consistently excluding patient rotation from recommended practice. This omission underscores the perception of rotation as a historically derived, non–evidence-based intervention rather than a validated component of intravesical therapy. The following section reviews the first randomized trial directly evaluating the clinical impact of patient rotation versus no rotation during intravesical BCG instillation.

A comparison of major international guideline positions regarding intravesical instillation technique is summarized in Table 1.

### Randomized Trial Data: Rotation vs. No Rotation

In 2023, Hariri et al^14^ reported the first randomized clinical trial directly comparing conventional “rotisserie” patient repositioning with no formal rotation during intravesical BCG instillation. In this single-center study, 52 patients with Ta/T1 NMIBC were randomized in a 1:1 fashion to either a rotation arm—undergoing 15-minute turns through supine, prone, left lateral, and right lateral positions during the first hour after instillation—or a no-rotation arm in which patients were discharged without prescribed positional changes. All patients received 120 mg of BCG in 50 mL of saline according to standard Southwest oncology group (SWOG) schedules and were instructed to retain the instillation for 2 hours.

At 1-year follow-up, recurrence rates were similar between groups, with no statistically significant difference observed between the rotation arm (26.9%) and the non-rotation arm (23.1%) (log-rank *P* = .82). While the study was not powered to detect modest differences in progression or long-term oncologic outcomes, these findings suggest that enforced patient rotation does not confer a measurable short-term recurrence benefit under standard BCG instillation conditions.

Importantly, the authors emphasized the pragmatic implications of omitting routine rotation, noting improved workflow efficiency, reduced patient discomfort, and the feasibility of same-day discharge without apparent compromise in early oncologic control.^14^ These observations align with prior expert commentary proposing that bladder wall apposition around relatively low instillation volumes, combined with incidental patient movement, is sufficient to achieve adequate mucosal exposure in the absence of intravesical air pockets.

Taken together, Hariri et al’s findings provide the only prospective comparative data available on this topic and offer supportive, though not definitive, evidence that routine rotation may be unnecessary in contemporary practice. Rather than serving as conclusive proof, this trial complements existing physiological rationale and guideline silence on rotation, reinforcing the view that mandated positional protocols represent a legacy practice lacking robust empirical validation. Larger studies with longer follow-up would be required to exclude small differences in recurrence or progression risk; however, current evidence does not support the routine enforcement of rotation during intravesical BCG therapy.

### Supporting Observations Against Rotation

Prior to the publication of the randomized trial by Hariri et al, indirect clinical evidence and expert commentary had already raised questions regarding the necessity of routine patient rotation during intravesical therapy. In a 2013 expert review, Shah and Kamat proposed that systematic repositioning is primarily relevant in the setting of a technical error—specifically, the introduction of an intravesical air bubble that may limit mucosal contact at the bladder dome. With contemporary instillation techniques that emphasize expelling air from syringes and catheters before drug delivery, such circumstances are uncommon, reducing the theoretical justification for scheduled rotation.^12^ In this context, physiologic bladder wall apposition with low-volume instillation is expected to permit broad mucosal exposure without formal positional maneuvers.

From a practical standpoint, informal reports from clinical practice have suggested that discontinuation of mandatory rotation does not appear to adversely affect short-term oncologic outcomes, while potentially improving patient tolerance. Intravesical BCG is frequently associated with urgency, dysuria, and bladder spasms, and enforced turning protocols may exacerbate discomfort during the dwell period. Allowing patients to remain in a self-selected, comfortable position—or to ambulate gently—has been associated with improved procedural tolerance, although these observations are derived largely from non-randomized experience rather than controlled comparisons.

In sum, physiologic reasoning, expert consensus, and real-world practice patterns were largely concordant with the findings of Hariri et al, suggesting that routine rotation is unlikely to be a critical determinant of intravesical therapy efficacy. Importantly, however, the available evidence base remains limited. While no randomized studies have specifically evaluated rotation during intravesical chemotherapy instillation (e.g., mitomycin-C or gemcitabine), differences in fluid properties and dwell logistics do not provide a compelling mechanistic rationale for greater reliance on positional maneuvers in these settings. As such, recommendations against routine rotation should be interpreted as consensus-driven and supported by indirect evidence, rather than definitive proof of equivalence across all agents and clinical contexts.

### Catheter Management: Removal vs. Clamping

Catheter management during the dwell period is an important procedural variable that may influence intravesical drug exposure, patient tolerance, and infection risk. Immediate catheter removal after instillation is commonly practiced and is supported by physiological rationale and expert consensus, although high-level comparative data are limited. Removal of the Foley catheter eliminates focal mucosal compression from the balloon, avoids catheter-associated microtrauma, and may reduce bladder spasms, thereby facilitating more uniform urothelial contact during the dwell period. Observational series and institutional reports suggest lower rates of catheter-associated urinary tract infection and improved patient comfort when the catheter is removed immediately following instillation, compared with prolonged clamping, although these findings have not been validated in randomized trials.^12^

Clamped Foley catheters are generally reserved for select patients who are unable to voluntarily retain the instilled volume, such as those with significant urinary incontinence or cognitive impairment. In these situations, best-practice recommendations include using the smallest feasible catheter caliber (typically 12 Fr), minimal balloon inflation (2-3 mL), and removing the catheter at the earliest clinically appropriate opportunity, ideally prior to discharge. While no randomized studies have directly compared oncologic outcomes or intravesical drug penetration between catheter removal and clamping strategies, available physiological considerations, expert opinion, and real-world practice patterns favor early catheter removal as a pragmatic, patient-centered approach rather than an evidence-proven mandate.

### Technical Nuances: Volume, Dwell, and Bladder Preparation

Instilled volume and dwell time are key determinants of intravesical therapy tolerability and effectiveness. Volumes in the range of 40-60 mL are widely used and appear sufficient to achieve comprehensive mucosal contact within a collapsed bladder while maintaining acceptable patient comfort. Higher volumes, particularly those exceeding 75 mL, are associated with increased urgency, leakage, and premature termination of dwell time, potentially undermining effective exposure.^10^ Computational and modeling studies suggest that larger volumes may modestly enhance transmural penetration; however, these theoretical gains must be balanced against reduced tolerability in clinical practice.

Recommended dwell times generally range from 60 to 120 minutes. Randomized data demonstrate improved efficacy of mitomycin C with a 1-hour dwell compared to shorter exposures, with no clear evidence of incremental benefit beyond 2 hours.^15^ Accordingly, many protocols target a dwell time of approximately 2 hours while accepting a minimum of 1 hour when patient tolerance is limited. Rigorous pre-procedure bladder emptying, achieved through fluid restriction and complete catheter drainage, remains an important preparatory step to prevent dilution and maintain consistent intravesical drug concentration, factors that are believed to contribute to optimal tumoricidal activity.

### Patient Repositioning

Converging lines of evidence suggest that routine patient rotation during intravesical therapy is unnecessary. The only randomized trial addressing this question, conducted by Hariri et al., demonstrated no significant difference in 1-year recurrence rates between patients undergoing a conventional rotisserie protocol and those managed without formal repositioning (26.9% vs. 23.1%; *P* = .82).^14^ While this study is limited by sample size and duration of follow-up, it provides the highest available level of comparative evidence directly evaluating the clinical impact of rotation.

These findings are consistent with earlier physiologic reasoning and expert commentary. Shah and Kamat previously noted that a collapsed bladder containing approximately 50 mL of instilled solution allows sufficient passive mucosal contact through bladder wall apposition, rendering scheduled positional changes unnecessary in the absence of technical errors such as air entrapment.^12^ In parallel, major professional guidelines—including those from the EAU, AUA/SUO, and CUA—do not recommend patient rotation during dwell time, implicitly reflecting the absence of supporting evidence.^8^^,^^9^^,^^11^

Collectively, available randomized data, expert interpretation, and guideline consensus support the omission of mandated patient repositioning during intravesical therapy. Patients may safely be instructed to retain the instilled solution for the prescribed dwell time while remaining in any comfortable position, including sitting, standing, ambulating, or lying supine, without compromising oncologic outcomes.

### Operational and Patient-Experience Benefits

De-implementation of mandatory patient rotation during intravesical therapy offers meaningful operational and patient-centered advantages. Eliminating enforced positional changes reduces chair time, facilitates same-day discharge in outpatient settings, and improves clinic workflow efficiency. From the patient perspective, avoidance of scheduled turning mitigates discomfort during an already irritative treatment and may improve adherence to induction and maintenance regimens.

By emphasizing technique elements supported by clinical data—appropriate dwell duration, meticulous bladder preparation, timely instillation, and judicious catheter management—clinicians can streamline intravesical therapy delivery while maintaining oncologic efficacy. These practical benefits further support reassessment of historically entrenched practices that lack empirical validation.

### Special Considerations

Although routine patient rotation is unnecessary in standard practice, select clinical scenarios may warrant individualized adjustments. In the uncommon event of inadvertent intravesical air instillation, simple postural changes such as standing or sitting may facilitate redistribution of the therapeutic solution toward the bladder dome. Similarly, patients with large or complex bladder diverticula may require extended dwell times or targeted maneuvers to enhance intraluminal exposure within outpouchings.

Importantly, these circumstances represent exceptions rather than indications for systematic rotation protocols, which remain unsupported for routine intravesical therapy delivery.

### Future Directions

Emerging innovations in intravesical drug delivery may further refine NMIBC management by reducing reliance on patient compliance and minimizing variability in instillation technique. Thermosensitive hydrogels that transition from liquid to gel at body temperature have demonstrated prolonged intravesical residence times and sustained urothelial exposure in preclinical bladder models.^16^ Similarly, nanoparticle-based formulations of BCG and chemotherapeutic agents have been explored as a means of controlled intravesical drug release, potentially extending therapeutic exposure while reducing peak-related toxicity.^17^ Drug-eluting platforms, adapted from ureteral and vascular stent technologies, represent another investigational approach to achieving sustained mucosal contact without repeated catheterization.^18^ While promising, these technologies remain largely preclinical or early-phase and require prospective validation before clinical adoption.

Advances in intravesical imaging may also inform future procedural optimization. Techniques such as intravesical fluorescence mapping and ultrasound-enhanced microbubble dispersion have been investigated as methods to visualize agent distribution and bladder wall contact in real time.^19^ If validated, such approaches could help identify suboptimal drug coverage related to bladder anatomy or instillation technique, thereby guiding individualized procedural adjustments rather than uniform protocols.

From a clinical research standpoint, there remains a need for well-designed, multicenter studies examining intravesical delivery parameters across agents beyond BCG. In particular, comparative trials evaluating catheter removal versus clamped Foley strategies for intravesical chemotherapy (e.g., mitomycin-C, gemcitabine) are warranted, incorporating oncologic outcomes, patient-reported tolerability, and health-economic endpoints. In parallel, remote monitoring strategies—including telehealth-based dwell-time tracking and patient-reported outcome tools—may offer pragmatic solutions to improve adherence and safety, especially in settings where prolonged in-clinic observation is impractical.^20^

### Tumor Grade, Risk Stratification, and Emerging Analytic Tools

Current NMIBC management frameworks rely primarily on tumor stage to guide treatment selection and surveillance intensity. While stage remains essential for anatomical classification and procedural planning, tumor grade reflects intrinsic biological aggressiveness and is a critical determinant of recurrence and progression risk. High-grade NMIBC, even when superficially staged, is associated with substantially worse oncologic outcomes compared with low-grade disease, underscoring the importance of accurate histopathologic assessment.^21,22^

Recent advances in digital pathology and computational analytics have generated interest in the use of artificial intelligence (AI) to augment traditional risk stratification. Early studies suggest that machine-learning models applied to histopathologic images, molecular profiles, and longitudinal clinical data may improve prediction of recurrence and progression beyond conventional clinicopathologic variables.^23^ At present, however, most AI-driven models in NMIBC remain investigational, with limited external validation and uncertain generalizability across practice settings.

Rather than replacing established staging systems, emerging analytic tools may ultimately complement existing frameworks by refining risk estimates and informing surveillance or treatment intensity. Future integration of such approaches will require rigorous prospective validation, transparent model development, and clear demonstration of clinical utility before incorporation into routine NMIBC care. Within this context, procedural optimization of intravesical therapy—grounded in evidence and patient-centered practice—remains a foundational component of effective bladder cancer management.

## Conclusion

Routine patient repositioning during intravesical treatment dwell time is not supported by available clinical evidence and does not appear necessary for effective drug distribution. Eliminating scheduled rotation simplifies workflow, improves patient comfort, and may enhance adherence without compromising oncologic outcomes.

Immediate catheter removal after instillation is a reasonable best-practice approach that may optimize urothelial exposure while reducing focal mucosal compression, bladder irritation, and catheter-associated complications. Clamped Foley catheters should be reserved for select patients unable to voluntarily retain intravesical agents and removed as early as clinically feasible.

Finally, while intravesical therapy optimization remains grounded in procedural technique, emerging advances in tumor grading, molecular characterization, and digital pathology, including cautiously applied AI tools—may further refine risk stratification and treatment selection in NMIBC. These developments should be viewed as complementary to established staging systems and warrant continued validation before routine clinical integration.

## Figures and Tables

**Table 1.** Guideline Positions on Intravesical Instillation Technique in Non–Muscle-Invasive Bladder Cancer

**Guideline Organization**	**Agent Selection and Scheduling**	**Dwell Time Recommendations**	**Patient Repositioning**	**Catheter Management**	**Key Notes Relevant to Technique**
EAU (European Association of Urology)	Recommends risk-adapted use of intravesical BCG and chemotherapy; emphasizes induction and maintenance schedules for high-risk NMIBC	Typically 1-2 hours depending on agent	Not addressed in physician guideline	Not explicitly specified	Focuses on oncologic efficacy and scheduling rather than procedural positioning
EAUN (European Association of Urology Nurses)	Supports guideline-based intravesical therapy delivery	Aligns with EAU dwell-time guidance	Explicitly states no evidence supports patient repositioning	Encourages patient comfort and safe handling	Notes inconsistency in manufacturer instructions (e.g., TICE vs. BCG Medac) and defaults to patient-controlled positioning
AUA/SUO (American Urological Association/Society of Urologic Oncology)	Endorses BCG and intravesical chemotherapy based on risk stratification	Recommends adequate dwell time (commonly ≥1 hour)	Not addressed	Not explicitly specified	Emphasizes safety, dosing, and risk stratification; omits technical positioning maneuvers
CUA (Canadian Urological Association)	Supports intravesical BCG and chemotherapy for appropriate risk groups	Aligns with standard dwell-time practices	Not addressed	Not addressed	Provides no recommendations on post-instillation positioning or catheter retention
Manufacturer Inserts (BCG formulations)	Product-specific dosing instructions	Typically recommend 1-2 hour retention	Inconsistent (e.g., TICE recommends rotation; BCG Medac advises ambulation)	Often imply catheter removal	Highlight lack of harmonization between manufacturers

BCG, Bacillus Calmette–Guérin; NMBIC, non–muscle-invasive bladder cancer.

## Data Availability

The data that support the findings of this study are available on request from the corresponding author.
